# Monthly Review of Parisian Medicine and Surgery

**Published:** 1859-09

**Authors:** Theophilus Mack

**Affiliations:** Lecturer on Materia Medica and Therapeutics in the Medical Department of the University of Buffalo


*Monthly Review of Parisian Medicine and Surgery:* By THEOPHILUS MACK, M. D., Lecturer on Materia Medica and Therapeutics in the Medical Department of the University of Buffalo.

At the "Academy of the Sciences," M. Cl. Bernard presented a Memorandum from Messieurs Demarquay and Leconte, upon the influence which gases exercise upon the healing of sub cutaneous wounds. "After having established the mean duration of the repair of tendons divided under the skin in rabbits, they have discovered, under the influence of daily injections of air within the wound, the work of organization is completed sensibly in the same time; injection of pure oxygen retards it, without producing inflammation. Hydrogen produces an inflammation accompanied by considerable abnormal vascularity, which occassions a great hindrance in the organization. Lastly, carbonic acid, on the contrary favors the organization of sub-cutaneous wounds, and brings about their cure in a much shorter time than in the tenotomies made without the influence of the air."

M. Flourens continued his communications of formation of bone by periosteum, with the view of demonstrating the forces which preside in the evolution of organic matter and decide *form*. This force he designates *morpha-plastic;* and he also attempts to prove the existence of another power, the meta-plastic, which effects its continual metamorphosis.

M. C. Rouget read an essay upon the part which the amylaceous matter styled by chemists *zoamyline,* performs in the evolution of embryonic tissue.

M. Petrequin, in treating of the application of electricity in vesical paralysis and catarrh, makes the subjoined excellent remarks:

"It will be seen that the employment of this or that mode of electrizing is not indifferent. Rigorous observation of the phenomena has led me to recognize that the dynamic action of the pile which acts upon the nervous system augments under the empire of the multiplications, and by the shocks which follow the production of sparks. The induction instruments, which furnish *volto-jara-daic* currents, realize the most convenient conditions for combating paralysis successfuly. Practically, it must not be forgotten, and the counsel of health of the armies insists with reason upon this recommendation, that if the electric current directed upon a nerve has only a moderate energy, it seems to replace or reinforce only the physiological action of the nerve in default; but that nevertheless, under too prolonged an influence of even moderate electric currents, the excitability ot the nerves is gradually enfeebled and may be even exhausted; that, on the other hand, every action of electric currents, to propagate itself tends to the whole nervous system and produce reflex effects; and these reflex effects are the more to be dreaded the more intense the currents are, &c. It is important in general to make the sitting short and to have recourse to electrization, temperate, and localized upon the nerves to be excited. Here is what anatomy teaches us as to those of the bladder: 'The nerves of the bladder are furnished by the *vesical plexus,* dependent on the hypogastric plexus, which itself emanates from the sacral plexus.—This last is formed by the pelvic portion of the great sympathetic, and by the vesical branches of the spinal, sacral nerves; which, united to the sacro-lumbar, terminate in the sciatic nerve. . . . The vesical pexus communicates with the hemorrhoidal plexus, another branch of the sacral plexus. Hence, physiologically, we are led to apply electricity in the treatment of paralysis of the bladder by placing one element of excitation in the bladder and the other in the rectum.' This has been our plan. Further, we have left urine in the bladder, instead of emptying it as practiced before, to the end that it may serve as a conducter to all the internal surface of the organ. Lastly we have, to act upon the anterior and top of the bladder, the current carried by an excitor to the centre of the hypogastrium.—We may add, that we should resort to it with reserve, avoid the reflex effects, which sacrcely ever fail to take place if we wander towards the root of the thighs or the iliac spines."

Vital statistics in France show the mortality of the human species to be greater between the ages of twenty and twenty-five, than between twenty-five and thirty. Dr. Bertillou explains this unexpected fact by the conscription, which subjects to the increased mortality of military life a large proportion of men from twenty to twenty-five.

ACADEMY OF MEDICINE.—A discustion upon the work of M. Labourdette relative to the introduction of medicaments into milk by assimilative digestion, led to the expression of some doubts as to the efficacy of this indirect medication, especially as to the superiority of iodized milk over cod-liver oil.

At the *séance* of the 10th May, further contributions were received as to the evils resulting from the rise and manufacture of arsenical green pigments, and the means of obviating those evils.

The Committee upon the contagiousness of secondary sphilis reports :

1st. That there are secondary syphilitic accidents manifestly contagious at the head of which they place the *flat tubercle*.

2d. This rule is applicable to nurses and suckling, as well as to other cases; and there is no reason to suppose that with infants at the breast, the results should be different from those in the adult.

In "The Medical Union" appears a paper from M. Laure, a naval surgeon, on Re Vaccination; he states.

1. That vaccine matter transferred from arm to arm is most efficacious.

2. Virus from re-vaccinated adults is as reliable as that taken from children.

3. Re-vaccination with adults should be performed more deeply than under the epidermis.

4. Re-vaccination is an important hygienic measure, the execution of which should be carefully watched.

5. This proceeding is as necessary with those who have suffered from variola, as with those who have been subject to cow-pox inoculation.

6. The local affection is slight in re-vaccination, provided rest be enjoined on the fifth day. The constitutional effects are important.

In-growing toe-nail is removed by M. Gourut by the following process; "Short strips of adhesive plaster are placed on one another, so as to form a kind of brim; two such fasciculi are made and placed a little in front and a little behind the morbidly growing nail. Into the groove thus artificially made, and which just occupies the root of the nail, semi-fluid Vienna paste is put. After a few minutes a black eschar is formed properly hemmed and limited by the adhesive plaster. The paste is now quickly taken off; and in a few days the nail comes off, without pain, by the gentlest traction.

M. Chalut reports the reduction of a case of strangulated hernia, by copious and frequently repeated draughts of strong coffee.

The treatment of chorea by arsenious acid, is spoken well of in the *Gaz. des Hôpitaux.*

Variolous orchitis is described in a clinical review in the *Gaz des Hôp.;* and it is demonstrated that variola has an influence upon the spermatic organs.

At the "Chirurgical Society," two observations were contributed, of successful treatment of hæmorrhage from the deep palmar arch, by compression of the humeral artery; and of hæmorrhage from the indicator, controlled by digital compression of both radial and ulnar arteries.

The prophylactic action of bromine in pseudo-membranous affections is advocated, apparently deserves trial.

M. Marjolin has directed attention to a circumstance which will have the effect of modifying the laws of amputations, viz: the elongation of the bones after the performance of amputation in very young subjects.

Two cases of prolonged gestation are communicated by Dr. Liegard, one of 308 days and one of more than 10 months.

Doctor Fournier proposed a new method of treatment of stricture of the urethra:

"After having ascertained by means of the catheter, the precise seat of the lesion, we mark a line with nitrate of silver, about one centimetre in front of the stricture; it is as a mark for the guidance of the patient. Every time that he experiences a desire to micturate, he should exert a pressure with his fingers upon this line, a pressure sufficient to prevent the jet of urine, impelled with force, from escaping by the meatus.

"This jet of urine should have for its ojects, to dilate during about 15 seconds all the portion of the canal situated behind the spot compressed. After 15 seconds he ceases to compress the canal, in order to permit the escape of this first jet of urine, to repeat the compression anew, and the same operation four or five times successively, according to the quantity of urine contained in the bladder.

"The patient ought to feel the necessity of urinating five or six times in the course of the day; and for this purpose, he should drink liberally of some diuretic tisane, of *chien-dent* (dogs-grass,) for instance. To favor the success of the treatment, I recommend a moderate exercise, a diet not full—and principally chosen from the vegetable kingdom; and two simple baths each weak.

"In directing the treatment which I have submitted in a succinct manner, I have seen an organic stricture two centimetres in length, situated between the bulbous and membranous portions, disappear. It had existed more than a year. The treatment ended three months since, and the cure continues still."

M. Verneuil reported a case of "Calculus of the nasal fossæ," taken at the *début* for a neuralgia, then for a necrosis of the bones of the nose. Attack painful, very intense, and intermittent. Lithotrity in four sittings. Expulsion of the rest of the concretion.—Cure followed by slight deformity of the nose.

M. Hervey de Chegion stated that he had met with a similar calculus in the dead subject.

A case of complete luxation of the eye, is reported in the "Journal of Medicine and Surgery:"

The column of water from a fire-engine struck violently upon the eyelids, in full force, and at a short distance from the engine.—They were thus forced strongly backwards; and, contracting under the double influence of shock and cold, they compressed the globe of the eye, and extended so as in a manner to enucleate it, leaving it hanging out, on the cheek, retained only by the motor muscles and optic nerve.

Reduction was easily effected; and after careful and appropriate treatment for about ten days, the functions and normal appearance were completely restored to the organ.

It is refreshing in a country where "the powers that be" seldom meddle with medicine except to prune it of both honor and emolument, to have it in our power to record an act of munificence on the part of the French government, which reflects the highest credit upon them.

Dr. Sturne, having fallen a victim to his philanthrophy in sucking out the secretions through a tracheotomy tube, in the trachea of a child, the *Gaz. Hebdom.* informs us that government have provided for the support and thorough education of the unfortunate gentleman's son ! !

This, with the appointment of M. F. Rouband to the well-paid office of Inspector of Min. Waters of Rougues and the grant of the Cross of the Legion of Honor to M. Debut, evinces the readiness of France to appreciate the services of science.—*New York & Buffalo Medical Journal.*

